# Tyrosinase Inhibitory Effect and Antioxidative Activities of Fermented and Ethanol Extracts of *Rhodiola rosea* and *Lonicera japonica*

**DOI:** 10.1155/2013/612739

**Published:** 2013-11-06

**Authors:** Yuh-Shuen Chen, Hua-Chian Liou, Chin-Feng Chan

**Affiliations:** ^1^Department of Food Science and Technology, Hungkuang University, Taichung 43302, Taiwan; ^2^Department of Applied Cosmetology, Hungkuang University, No. 1018, Section 6, Taiwan Boulevard, Shalu District, Taichung 43302, Taiwan

## Abstract

This is the first study to investigate the biological activities of fermented extracts of *Rhodiola rosea* L. (Crassulaceae) and *Lonicera japonica* Thunb. (Caprifoliaceae). *Alcaligenes piechaudii* CC-ESB2 fermented and ethanol extracts of *Rhodiola rosea* and *Lonicera japonica* were prepared and the antioxidative activities of different concentrations of samples were evaluated using *in vitro* antioxidative assays. Tyrosinase inhibition was determined by using the dopachrome method with L-DOPA as substrate. The results demonstrated that inhibitory effects (ED_50_ values) on mushroom tyrosinase of fermented *Rhodiola rosea*, fermented *Lonicera japonica*, ethanol extract of *Lonicera japonica,* and ethanol extract of *Rhodiola rosea* were 0.78, 4.07, 6.93, and >10 mg/ml, respectively. The DPPH scavenging effects of fermented *Rhodiola rosea* (ED_50_ = 0.073 mg/ml) and fermented *Lonicera japonica* (ED_50_ = 0.207 mg/ml) were stronger than effects of their respective ethanol extracts. Furthermore, the scavenging effect increases with the presence of high content of total phenol. However, the superoxide scavenging effects of fermented *Rhodiola rosea* was less than effects of fermented *Lonicera japonica*. The results indicated that fermentation of *Rhodiola rosea* and *Lonicera japonica* can be considered as an effective biochemical process for application in food, drug, and cosmetics.

## 1. Introduction

Oxidative stress has been demonstrated to cause atherosclerosis, diabetes mellitus, aging, and even cancer [1]. It has been reported that sufficient intake of antioxidants, such as green tea, may be necessary to repress oxidative stress and prevent disease and aging [2].

Tyrosinase, a copper-containing polyphenol oxidase, plays a highly critical role in forming melanin pigments [3]. Previous reports have shown that tyrosinase might also be involved in neuromelanin production and be associated with Parkinson's disease [4]. Therefore, inhibiting tyrosinase activity is applicable to skin-lightening and in preventing neurodegeneration [4]. A broad spectrum of potent tyrosinase inhibitors, including ascorbic acid and its derivatives [5], thiol-containing amino acids (such as cysteine) [5], and different classes of phenolic compounds [3, 6], have been obtained from natural products [3].

This study investigated the tyrosinase inhibitory effect of two herbal medicines, *Rhodiola rosea* L. (Crassulaceae) and *Lonicera japonica* Thunb. (Caprifoliaceae). *Rhodiola rosea* exhibits various biological activities, such as antioxidative, antidepressant, anticancer, and neuroprotective effects [7, 8]. *Lonicera japonica* is used to treat exopathogenic wind-heat, epidemic febrile diseases, sores, neurodegeneration, and infectious diseases [9, 10]. In the past few years, fermented preparation of natural products has been proven to be useful in promoting gastrointestinal health and skincare [11], protecting against ethanol-induced toxicity [12], and enhancing immune activity [13]. However, no previous studies have investigated the biological effects of fermented *Rhodiola rosea* and *Lonicera japonica*. To promote the processing of these two herbs, this study developed a process for fermenting *Alcaligenes piechaudii *CC-ESB2 [14], wherein the functionality of the herbs is preserved and enhanced. Therefore, natural products have the potential to be further developed into novel functional foods, cosmetic raw material, and effective phytomedicine.

## 2. Materials and Methods

### 2.1. Materials

Tyrosinase, kojic acid, and L-DOPA were purchased from Sigma Chemical Co. (St. Louis, MO). Plant materials of *Rhodiola rosea* and *Lonicera japonica* were purchased from Jin Wan Biotechnology Co. Ltd. (Taichung, Taiwan). All the plant materials were cultivated in Guizhou, China. The plant materials were fresh harvested and lyophilized by Guizhou Sanxin group Co. Ltd. (Guizhou, China). The voucher specimens were further confirmed by the Approval and Certification Center of Association of Taiwan Tea, Taichung, Taiwan. The plant materials were ground into a powder before use.

### 2.2. Microorganism and Fermentation

*Alcaligenes piechaudii *CC-ESB2 are diesel-degrading strains [14]. Enrichment of *A. piechaudii* CC-ESB2 was conducted using a modified Bushnell's method [14, 15]. Briefly, 5 × 10^6^ of colony forming unit (CFU) bacteria were cultured with 0.5% *Rhodiola rosea* or *Lonicera japonica* in flasks with sand, 0.2% oil, and 500 mL BH medium at 30°C and cultured with shaking at 160 rpm under aerobic conditions for five days. The BH medium formula (g/L) is composed of K_2_HPO_4_: 1, KH_2_PO_4_: 1, NH_4_NO_3_: 1, MgSO_4_·7H_2_O: 0.2, CaCl_2_·2H_2_O: 0.02, and FeCl_3_: 0.05. After fermentation, the supernatant of broth was filtered and extracted twice using an equal volume of ethyl acetate. Hexane was used to extract and remove the oil of the sample. For ethanol extracts, both herbal medicines were twice extracted using 50% ethanol (W : V = 1 : 3). The samples were concentrated in a vacuum concentration device (PANCHUM R-2000V) and then freeze-dried (Freeze Dryer-FD-series, PANCHUM). The freeze-dried powder was stored at −20°C before use. The samples were reconstituted with 10% dimethyl sulfoxide (DMSO). The final concentration of DMSO in the reaction mixtures is less than 1%. One percent of DMSO was used as a vehicle control in the following assays.

### 2.3. Tyrosinase Activity Assay

Tyrosinase activity was determined as previously reported [16] with the modification of using a microplate. Briefly, in a 96-well plate, 20 *μ*L of 400 U of mushroom tyrosinase was mixed with 20 *μ*L of 0.1–10 mg/mL of fermented and ethanol herbal extracts. After 160 *μ*L of L-DOPA 0.5 mM was added to each solution, the solutions were monitored for the formation of dopachrome for 30 min by measuring the optical densities at 475 nm using an ELISA reader (TECAN, Austria) at room temperature. All experiments were conducted in triplicate. The tyrosinase inhibition rate was calculated as the inhibition rate (%) = 1 − (*A*_Sample  475 nm_/*A*_Control  475 nm_) × 100. The ED_50_ value was determined by regression of a constructing dose response curve at which 50% target activity was lost. 

### 2.4. Total Phenolic Content

The total phenolic content was determined by Liao's method [17]. A volume of 0.3 mL of samples at a concentration of 0.5 mg/mL was mixed with 2.4 mL of distilled water and 0.3 mL Folin-Ciocalteu reagent. Sodium carbonate (20%, 0.6 mL) was added to the reaction mixture and allowed to stand for 30 min. The absorbance at 730 nm was measured and compared to a gallic acid calibration curve and expressed as mg of gallic acid per gram of sample (GAE).

### 2.5. DPPH Free Radical-Scavenging Assay

The scavenging activity of sample extracts on 1,1-diphenyl-2-picrylhydrazyl (DPPH) radical was determined using a method previously described [17]. Fifty microliters of different concentrations (0.01, 0.02, 0.1, 0.2, 0.5, and 1 mg/mL) of ethanol or fermented extracts were mixed with 150 *μ*L of freshly prepared 0.1 mM DPPH in ethanol. DPPH absorbance was then measured at 517 nm, using an ELISA reader (TECAN, Austria). Each test was carried out in triplicate. Percent activity was calculated using the following equation:

(1) $$\begin{array}{c} \text{Activity}\;\left(\%\right)=1-\left(\frac{A_{\text{Sample}}}{A_{\text{Blank}}}\right)\times 100. \end{array}$$

### 2.6. Superoxide Radical-Scavenging Assay

Superoxide anion-scavenging ability was measured using a previously described method [17]. The phenazine methosulfate-nicotinamide adenine dinucleotide (PMS-NADH) system generates superoxide radicals, which reduce nitroblue-tetrazolium (NBT) to a purple diformazan compound. Briefly, reaction solutions containing various concentrations of herbal samples (50 *μ*L, 0.1 mg/mL to 2 mg/mL) mixed with PMS (80 *μ*M), NADH (1248 *μ*M), and NBT (200 *μ*M) in phosphate buffer (0.1 M, pH 7.4) were incubated at room temperature for 5 min. The color was read at 560 nm against blank samples. 1% DMSO was used as a vehicle control. The superoxide anion radical-scavenging percentage was calculated using the following equation:

(2) $$\begin{array}{c} \text{Scavenging}\;\text{effect}\;\left(\%\right)=1-\left(\frac{A_{\text{Sample}\;560\;\text{nm}}}{A_{\text{Control}\;560\;\text{nm}}}\right)\times 100. \end{array}$$

### 2.7. Statistical Analysis

Three samples were prepared for each assay. The results were expressed as mean and standard deviation. Data analysis included one-way ANOVA, followed by duncan's multiple range test (*P* < 0.05) and a correlation test using the SigmaStat 3.5 software program.

## 3. Results and Discussion

### 3.1. Tyrosinase Activity Assay

**Figure 1:**
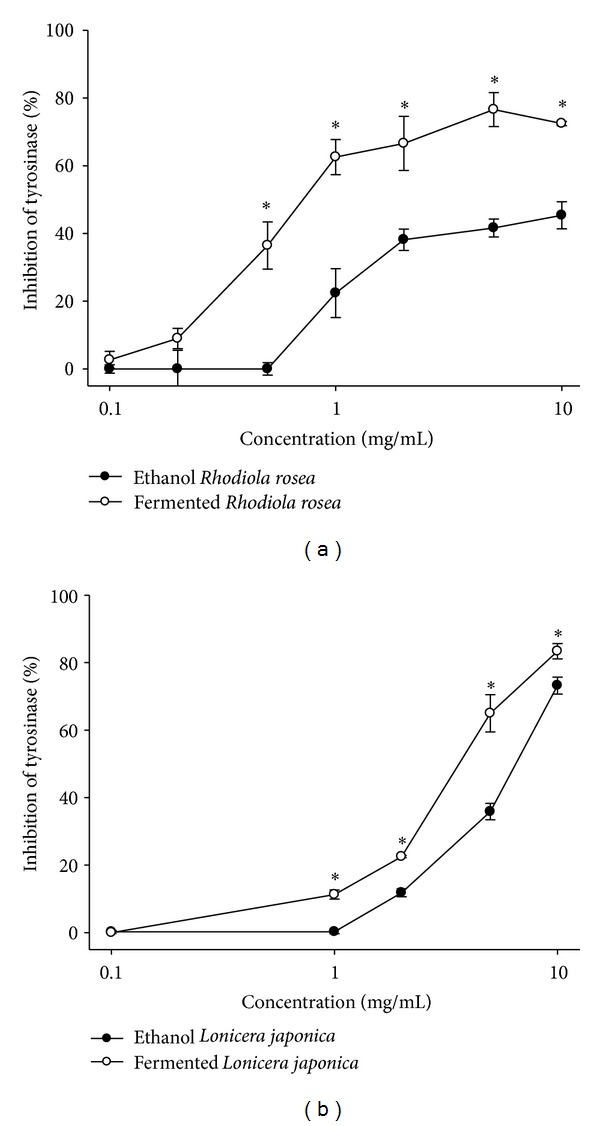
Tyrosinase inhibitory effects of *Rhodiola rosea *and *Lonicera japonica*. (a) Tyrosinase inhibitory effects of fermented and ethanol extracts of *Rhodiola rosea.* (b) Tyrosinase inhibitory effects of fermented and ethanol extracts of *Lonicera japonica*. Values are means ± SD (*n* = 3). Asterisk indicates a significant difference (*P* < 0.05) when compared to the data of fermented extracts with the value of respective ethanol extracts.

In this study, fermented extract of *Rhodiola rosea* exhibited potent inhibitory effect on tyrosinase activity in a dose response manner—the inhibitory effect was significantly greater than that of ethanol extract (Figure 1(a)). The ED_50_ of fermented *Rhodiola rosea* on the tyrosinase inhibitory effect is 0.78 mg/mL, which was about 12 times more potent than that of ethanol extracts (ED_50_ > 10 mg/mL) (Table 1). 

Fermented extract of *Lonicera japonica* also exhibited a higher inhibitory effect on tyrosinase activity, compared to the ethanol extracts (Figure 1(b)). The ED_50_ of fermented *Lonicera japonica* on the tyrosinase inhibitory effect is 4.07 mg/mL, which increases about 70% insensitive to ethanol extracts of *Lonicera japonica* (ED_50_ = 6.93 mg/mL) (Figure 1(a)). However, fermented extract of *Lonicera japonica* was less effective than fermented extract of *Rhodiola rosea* on the tyrosinase inhibitory effect.

In this study, ethanol extracts of *Rhodiola rosea* exhibited a high tyrosinase inhibition effect, which is consistent with the results of a previous report [18]. Furthermore, our results demonstrate that fermented extract of *Rhodiola rosea* has a more pronounced inhibitory effect on tyrosinase activity than does the effect of ethanol extract. Similar to gamma irradiation treatment [19], fermented treatment of *Lonicera japonica* exhibited higher tyrosinase inhibitory effects than did the effects of organic solvent extracts.

### 3.2. Total Phenolic Content

Among the four herbal extracts, fermented extract of *Rhodiola rosea* had the highest total phenolic content (241.58 ± 16.16 mg GAE/g sample), followed by ethanol extract of *Rhodiola rosea* (142.12 ± 10.27 mg GAE/g sample), fermented extract of *Lonicera japonica* (116.70 ± 3.96 mg GAE/g sample), and ethanol extract of *Lonicera japonica* (67.25 ± 9.91 mg GAE/g sample) (Table 1). This study also demonstrated that the total phenolic content of fermented extracts of *Rhodiola rosea* or *Lonicera japonica* was higher than the content of ethanol extracts of *Rhodiola rosea* or *Lonicera japonica*. The results were consistent with the previous reports that Bokbunja fermented by yeast had a higher total phenolic content than did the unfermented material [20].

### 3.3. DPPH Free Radical-Scavenging Assay

**Figure 2:**
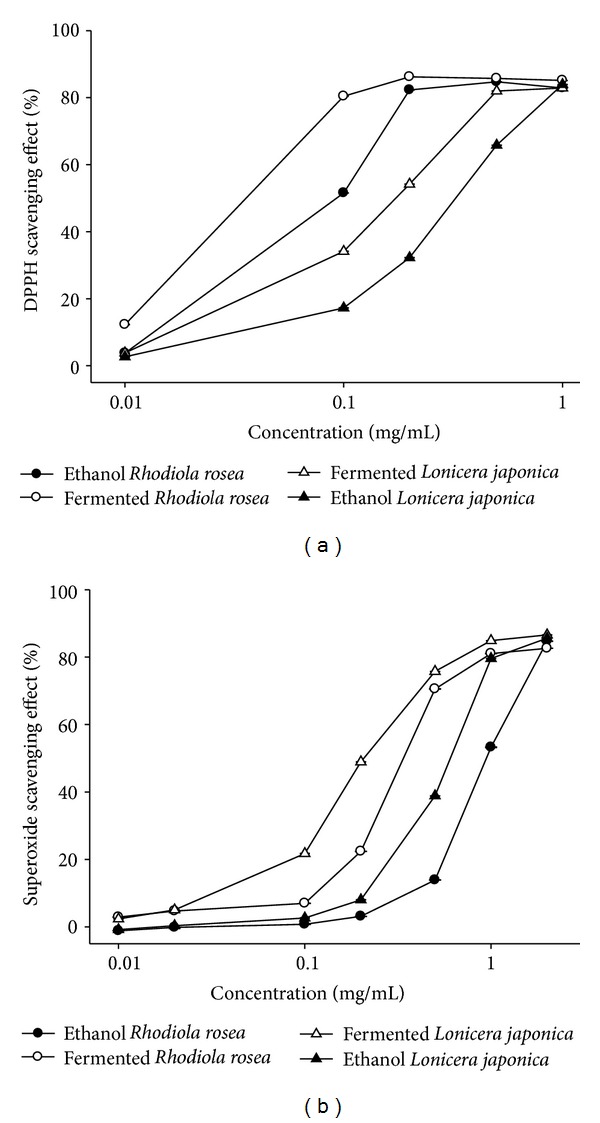
Antioxidative activities of *Rhodiola rosea *and *Lonicera japonica*. (a) DPPH radical scavenging effects of fermented and ethanol extracts of *Rhodiola rosea *and *Lonicera japonica.* (b) Superoxide scavenging effects of fermented and ethanol extracts of *Rhodiola rosea *and *Lonicera japonica*. Values are means ± SD (*n* = 3).

The DPPH radical-scavenging activity of four oils increased in a dose-dependent manner at sample concentrations between 0.01 and 1 mg/mL (Figure 2(a)). The results indicate that the DPPH radical-scavenging activity of *Rhodiola rosea* and *Lonicera japonica* reached a saturation point at concentrations of 0.2 and 0.5 mg/mL, respectively (Figure 2(a)). The ED_50_ of DPPH radical-scavenging activity of the 4 oils was inversely proportional to the order of total phenolic content (Table 1). The current results indicate that DPPH radical-scavenging activity was inversely correlated with total phenolic content (*r* ^2^ = 0.72).

### 3.4. Superoxide Radical-Scavenging Assay

Fermented extract of *Rhodiola rosea* exhibited potent superoxide radical-scavenging effect in a dose-dependent manner and possessed a significantly higher scavenging effect, as compared to the effect of ethanol extract (Figure 2(b)). The ED_50_ of fermented *Rhodiola rosea* is 0.37 ± 0.00 mg/mL; however, the ED_50_ of ethanol extracts of *Rhodiola rosea* is 1.15 ± 0.01 mg/mL. The ED_50_ of fermented and ethanol extracts of *Lonicera japonica* is 0.28 ± 0.01 mg/mL and 0.66 ± 0.00 mg/mL, respectively (Figure 2(b)). The order of superoxide radical-scavenging effect is fermented extract of *Lonicera japonica*  > fermented extract of *Rhodiola rosea* > ethanol extract of *Lonicera japonica* > ethanol extract of *Rhodiola rosea*. 

The results also demonstrate that 2 herbs fermented by *Alcaligenes piechaudii *CC-ESB2 exhibit a considerably more potent effect on superoxide radical-scavenging than do the effects of ethanol extracts (Figure 2(b)). Surprisingly, in contrast to tyrosinase inhibitory and DPPH scavenging effects, the superoxide radical-scavenging effect of *Lonicera japonica* was more favorable than the effect of *Rhodiola rosea* (Figure 2(b)). The results may be due to *Lonicera japonica* [21] having more active components of superoxide radical-scavenging activity than does *Rhodiola rosea* [22]. It has been reported that *Lonicera japonica* extracts from flowers and leaves exerted potent superoxide radical-scavenging activities with ED_50_ values 16.60 and 38.63 *μ*g/mL, respectively [23]. 

## 4. Conclusions 

The investigated fermented *Rhodiola rosea* and *Lonicera japonica* have richer secondary metabolites such as phenolic contents compared to ethanol extracts of *Rhodiola rosea* and *Lonicera japonica*. Therefore, due to their high active components, fermentation can be considered as an effective biochemical process for application in food, drug, and cosmetics. Particularly, the expected potential of fermented *Rhodiola rosea* as a skin whitening and antiaging agent strengthens the possibility of its commercial use in food, drugs, and cosmetics. A previous report demonstrated that fermented *Radix astragali* exhibited more effects on hyaluronic acid (HA) production in primary human skin cells than did the effect of unfermented *Radix astragali* [11]. Investigation of the effects of fermented *Rhodiola rosea *and *Lonicera japonica* extracts on the collagen synthesis and hyaluronic acid production in primary human skin cells will be conducted in future study.

## Figures and Tables

**Table 1:** Total phenolic content and IC_50_ values of antioxidative activities of fermented and ethanol extracts of *Rhodiola rosea* and *Lonicera japonica*.

	*Rhodiola rosea *		*Lonicera japonica *	
	Ethanol	Fermented	Ethanol	Fermented
Total phenolic content (mg GAE/g sample)	142.12 ± 10.27^b#^	241.58 ± 16.16	67.25 ± 9.91^d^	116.70 ± 3.96^c^
DPPH scavenging ED_50_ (mg/mL)	0.11 ± 0.00^b^	0.07 ± 0.00^a^	0.45 ± 0.01^d^	0.21 ± 0.01^c^
Superoxide scavenging ED_50_ (mg/mL)	1.15 ± 0.02^d^	0.37 ± 0.00^b^	0.66 ± 0.01^c^	0.28 ± 0.00^a^

^#^The samples with different letters in superscript in the same assay indicate a significant difference (*P* < 0.05).
